# Optical Performance Assessment of Nanostructured Alumina Multilayer Antireflective Coatings Used in III–V Multijunction Solar Cells

**DOI:** 10.1021/acsaem.2c00133

**Published:** 2022-04-25

**Authors:** Jarno Reuna, Arttu Hietalahti, Arto Aho, Riku Isoaho, Timo Aho, Marianna Vuorinen, Antti Tukiainen, Elina Anttola, Mircea Guina

**Affiliations:** Optoelectronics Research Centre, Physics Unit, Faculty of Engineering and Natural Sciences, Tampere University, P.O. Box 692, FI-33014 Tampere, Finland

**Keywords:** antireflective coating, nanostructuring, III−V multijunction solar cell, omnidirectional, broadband

## Abstract

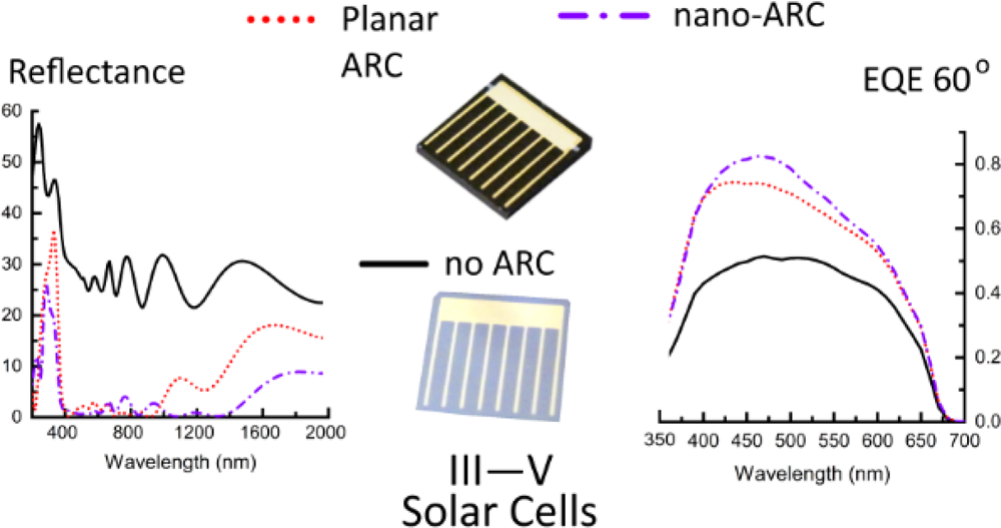

The optical performance of a multilayer antireflective coating incorporating lithography-free nanostructured alumina is assessed. To this end, the performance of single-junction GaInP solar cells and four-junction GaInP/GaAs/GaInNAsSb/GaInNAsSb multijunction solar cells incorporating the nanostructured alumina is compared against the performance of similar solar cells using conventional double-layer antireflective coating. External quantum efficiency measurements for GaInP solar cells with the nanostructured coating demonstrate angle-independent operation, showing only a marginal difference at 60° incident angle. The average reflectance of the nanostructured antireflective coating is ∼3 percentage points smaller than the reflectance of the double-layer antireflective coating within the operation bandwidth of the GaInP solar cell (280–710 nm), which is equivalent of ∼0.2 mA/cm^2^ higher current density at AM1.5D (1000 W/m^2^). When used in conjunction with the four-junction solar cell, the nanostructured coating provides ∼0.8 percentage points lower average reflectance over the operation bandwidth from 280 to 1380 nm. However, it is noted that only the reflectance of the bottom GaInNAsSb junction is improved in comparison to the planar coating. In this respect, since in such solar cells the bottom junction typically is limiting the operation, the nanostructured coating would enable increasing the current density ∼0.6 mA/cm^2^ in comparison to the standard two-layer coating. The light-biased current–voltage measurements show that the fabrication process for the nanostructured coating does not induce notable recombination or loss mechanisms compared to the established deposition methods. Angle-dependent external quantum efficiency measurements incline that the nanostructured coating excels in oblique angles, and due to low reflectance at a 1000–1800 nm wavelength range, it is very promising for next-generation broadband multijunction solar cells with four or more junctions.

## 1 Introduction

High efficiency III–V multijunction solar cells (MJSC) offer the most advanced photovoltaic technology to date, with the highest confirmed conversion efficiency reaching 47.1%^1,2^ and theoretical efficiency surpassing 50%.^3−5^ Such MJSCs utilize a very broadband spectrum of the solar irradiation, and significant losses can come from the reflected light from the surface of the cell. Conventional double-layer antireflective coatings (ARC) have been frequently used in MJSC applications,^6−8^ but when exceeding three junctions, the current matching starts to require broader reduction of reflectance.^6,9^ This is especially true for solar cell structures with a germanium bottom junction, where the usable spectral bandwidth extends up to 1800 nm.^10−12^ In general, different kinds of nanostructured ARCs have been applied in order to obtain low reflectance in a broad spectral band,^13−20^ but they typically come with their drawbacks. With a patterned semiconductor window layer, there are additional losses in the ultraviolet region due to the need for thick window layers,^15−17^ direct patterning of the solar cell structure can cause increased recombination losses,^18^ and with patterned dielectric structure, the refractive index contrast between the high index semiconductor material and the low index ARC is too large for efficient reduction of reflectance.^13,14^ Multilayer dielectric coatings combined with patterning^9,19,20^ have so far been an effective but laborious solution due to multistep fabrication processes.

As an alternative, we proposed recently^21^ a simple, nontoxic, low-cost nanostructured multilayer ARC that is based on postdeposition treatment of planar amorphous alumina coatings with heated deionized water.^22,23^ An advantage of this approach compared to similar kind of hybrid broadband ARC^19,20^ is the reduced number of fabrication steps, as it does not need lithography and surface etching for patterning. The properties of the nanopattern can be controlled via film thickness and treatment time,^24^ and the hydrophobicity of the film can be increased with postprocess polymerization.^25^

Here, we present a comparison between the performance of the novel nanostructured ARC and conventional planar ARC when applied to single-junction (1J) and four-junction (4J) III–V solar cells (SC). The 1J structure is used for assessing the angle-dependent characteristics of the nanostructured coating, as with MJSC such characterization is challenging to do correctly and requires more developed instrumentation.^26,27^ The actual broadband operation and suitability for MJSCs are then verified with the 4J SCs.

## 2 Experimental Section

The lattice-matched III–V SC structures, namely, GaInP 1J and GaInP/GaAs/GaInNAsSb/GaInNAsSb 4J, with band gap energies of 1.9 eV/1.4 eV/1.2 eV/0.9 eV, respectively, were grown by molecular beam epitaxy on p-GaAs substrates using a Veeco GEN20 MBE system. Specific structural details and the performance of the reference cells are provided elsewhere.^7^ The wafers were diced into 6 mm × 6 mm SCs with an active area of 0.25 cm^2^. Both the Ni/Au (10/100 nm) front contact grid on the n-side and Ti/Au (50/100 nm) planar back contact on the p-side were deposited using an electron beam (e-beam) evaporator. Prior to ARC deposition, the contact GaAs layer was removed with NH_3_/H_2_O_2_/H_2_O etchant solution.

**Figure 1:**
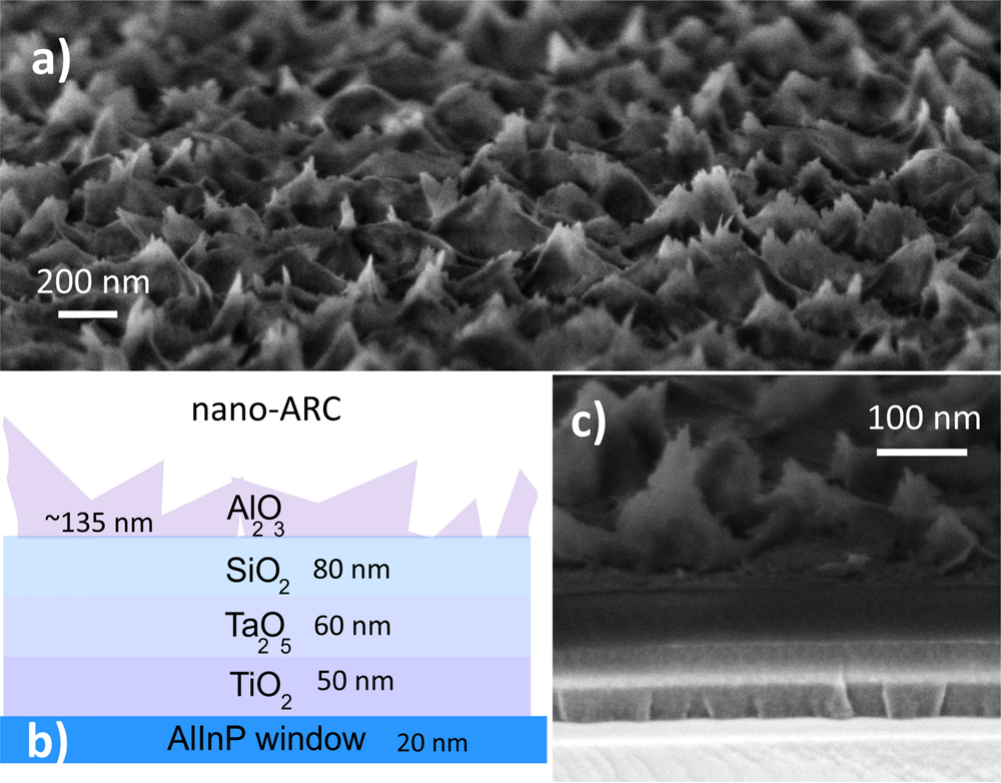
(a) Scanning electron microscope surface scan of the nano-ARC, (b) schematic illustration of its structure, and (c) cross-sectional scanning electron micrograph of the nanostructured coating.

The conventional planar double-layer ARC was grown by an e-beam, and the multilayer film for the nanostructured ARC (nano-ARC) was deposited by ion beam sputtering (IBS) using a Navigator 700 sputtering system from Cutting Edge Coatings GmbH. The nanostructuring of the amorphous alumina layer was achieved by treating the coating with heated deionized water (DIW). The method for nanostructuring the alumina surface is described in detail in reference.^21^ The nominal structure of the nano-ARC and its cross-sectional scanning electron microscope image are shown in Figure 1. The planar double-layer coating had the nominal structure of 50 nm TiO_2_/89 nm SiO_2_. The planar ARC has originally been optimized for the GaInP/GaAs/GaInNAsSb triple-junction MJSC,^28^ so that the top-junction (GaInP) would not be the current-limiting junction. It exhibits relatively broadband low reflectivity at 400–1000 nm and with the given materials represents a robust and realistic optimal double-layer ARC for these III–V MJSCs. This makes it a suitable comparison structure for the nano-ARC in question.

Scanning electron microscope (SEM) images were taken with a ΣIGMA FESEM operated with SmartSEM software, both products of Carl Zeiss NTS Ltd. The acceleration voltage was 1 kV, and the aperture size was 10 μm. The dielectric nature of the coating causes charging of the scanned area, which can cause image distortions. The imaged samples were tilted ∼10° in attempts to avoid areal charge accumulation.

A PerkinElmer Lambda 1050 UV/vis/NIR spectrophotometer equipped with either an integrating sphere or a Universal Reflectance Accessory (URA) module was used for the reflectance measurements. The URA measures reflectance at 8° angle of incidence and the integrating sphere at normal incidence. The URA module can measure only specular reflectance, whereas the integrating sphere nominally measures also all the scattered light. No notable difference in sample performance was observed between the modules, indicating negligible diffuse scattering from the nanostructure. For the spectrum-weighted average, the values were calculated as follows:
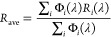
1in which Φ_*i*_ is the incident photon flux and *R_i_* is the measured reflectance at a given wavelength.

Light-biased current–voltage (LIV) characteristics of the SCs were measured with a 7 kW OAI Trisol solar simulator calibrated for AM1.5D (1000 W/m^2^) illumination. Evaluated properties include conversion efficiency η, open-circuit voltage *V*_OC_, short-circuit current density *J*_SC_, and fill factor FF. All the samples were measured at the same time, the measurements were repeated a number of times, and the average standard deviations for the quantities are 0.1 percentage points, 3 mV, 0.1 mA/cm^2^_,_ and 1 percentage points, respectively. In addition of the statistical uncertainties, the unideal spectrum of the used simulator lamp, which is known to be infrared-weighted, and temporal variations increase the error limits for drawing conclusions. The external quantum efficiency (EQE) measurements for the GaInP cells were performed with a monochromator-based measurement system, which was adjusted using a NIST (National Institute of Standards and Technology)-calibrated Si reference cell at room temperature (22 °C). An angle-selective stage (Thorlabs High-Precision Rotation Stage PR01/M) was used to accurately (±1°) align the incidence angle of the probe beam on the GaInP cells at variable angles from 0° to 45° and 60° to assess the oblique angle performance of the ARCs.

A python script based on May et al integration tools^29^ was used to calculate the ideal and EQE-derived current densities of different subjunction bandwidths according to both AM0 (ASTM E-490) and AM1.5D (ASTM G-173-03) solar spectra.^30^ In the calculations, the ARCs are assumed to be lossless (*T* = 1-*R*) and the internal quantum efficiency (IQE) to be unity. The cases where IQE = 1 and EQE = 1-*R* are labeled as ideal and represent the theoretical maximum when all the incident photons that are not reflected from the SC are absorbed and each generates a charge-carrier pair. This provides a comparable quantity representing the current-generation potential of different spectral bandwidths when assessing the differences of the ARCs. The most common applications of III–V multijunction SC materials are either in space or in terrestrial concentrator photovoltaics. AM0 spectra are the standard that is used for comparing space photovoltaic SCs used for instance in satellites. Similarly, AM1.5D is used for comparing the performance of III–V concentrator SC materials, as only direct sunlight can be efficiently concentrated. Comparing both gives a realistic evaluation of the nano-ARC performance in the possible applications.

## 3 Results and Discussion

**Figure 2:**
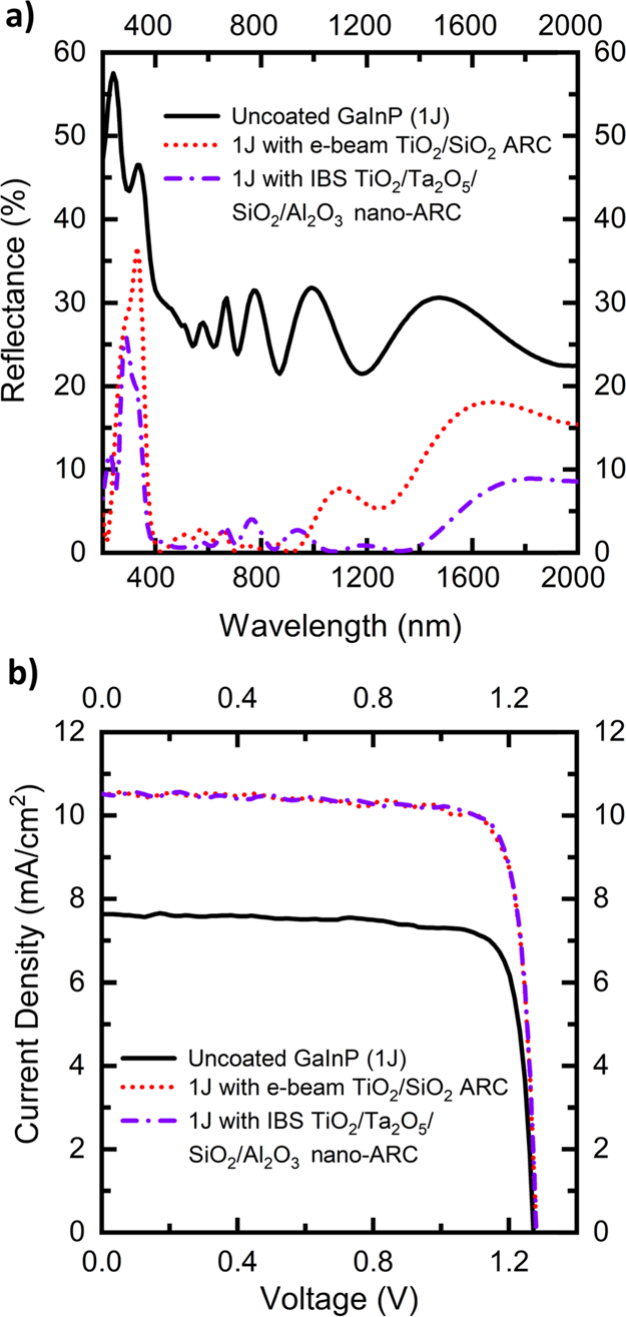
(a) URA-measured reflectance for uncoated GaInP 1J, with the conventional e-beam double-layer ARC and with the nano-ARC. (b) Measured LIV under AM1.5D (1000 W/m^2^) for the 1 J GaInP solar cells without a coating, with the e-beam double-layer ARC and with the nano-ARC.

The reflectance and LIV characteristics of the GaInP 1J cells with the nano-ARC and the planar double-layer ARC under AM1.5D (1000 W/m^2^) illumination are presented in Figure 2.

Figure 2a clearly shows that the nano-ARC has lower reflectance over broader bandwidths than the double-layer ARC, as was expected. The spectrum-weighted average reflectance values (*R*_AVE_) at the operative bandwidth of the GaInP SC are presented in Table 1. Based only on the reflectance values, it is expected that the GaInP SC with the nano-ARC should have better LIV performance.

**Table 1:** Spectrum-Weighted *R*_AVE_ for the Coated GaInP 1J Solar Cells Presented at the Bandwidth of Operation Both with AM0 and AM1.5D Spectra, and the Measured LIV Characteristics under AM1.5D (1000 W/m^2^) Illumination, with Conversion Efficiency η, Open-Circuit Voltage *V*_OC_, Short-Circuit Current Density *J*_SC_, and Fill Factor FF for Bare SC, with Planar e-Beam ARC, and with Nano-ARC

		bandwidth (nm)	uncoated	e-Beam TiO_2_/SiO_2_	IBS TiO_2_/Ta_2_O_5_/SiO_2_/Al_2_O_3_ nano-ARC
*R*_AVE_	AM0	280–710	28.8%	3.6%	2.2%
	AM1.5D	280–710	27.8%	2.3%	1.5%
η (%)			8.4	11.1	11.4
*V*_OC_ (V)			1.3	1.3	1.3
*J*_SC_ (mA/cm^2^)			8.0	10.9	10.8
FF [%]			81.7	79.2	82.6

The modest performance of the GaInP SCs in terms of efficiency and current density is due to the fact that the SCs in question are designed to be current matched as a part of an MJSC and not to be standalone SCs, thus being thinner than conventional junctions. The reasoning and the effects of thinning are further discussed elsewhere.^7,31^ However, as a topmost junction in MJSC configuration, they suit very well as ARC reference samples when the coatings are evaluated against each other.

**Figure 3:**
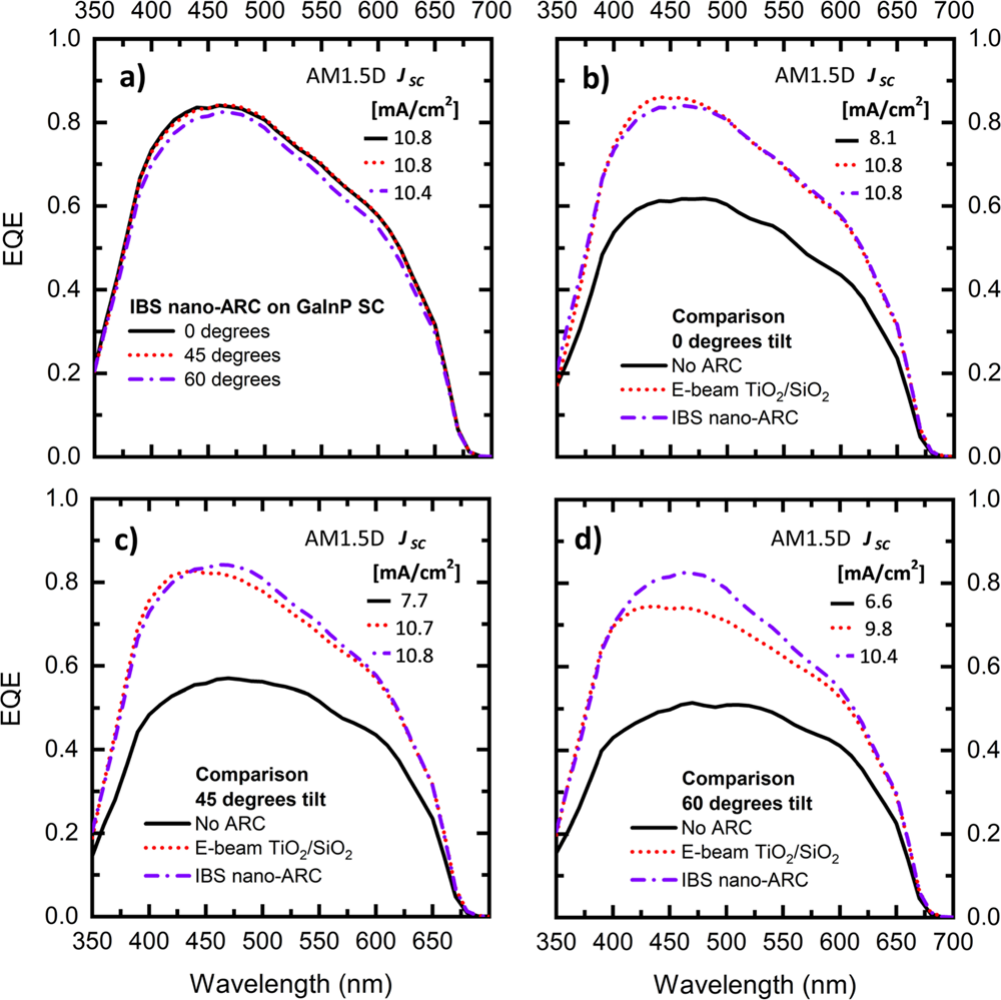
(a) Measured angle-dependent EQEs for the 1J GaInP solar cells coated with the nano-ARC at the angles of 0°, 45°, and 60°, demonstrating nearly unchanged performance as a function of the incidence angle. (b)–(d) Comparison of 1J GaInP solar cell EQEs without an ARC, with the e-beam ARC, and with the nano-ARC at the angles of 0°, 45°, and 60°, respectively. For each of the measured EQEs, the corresponding current density under the AM1.5D (1000 W/m^2^) spectrum has been calculated and is shown in the inset tables on the right upper corner of each subplot.

To see if nanostructuring has the expected^20,32^ angle-independent nature, the EQEs of the GaInP SC were measured at different angles of 0°, 45°, and 60°. The angle-dependent EQEs are shown in Figure 3, and the related calculational current densities under AM1.5D (1000 W/m^2^) are shown in inset tables for each subplot.

At the normal incidence angle, the EQEs of the coated SCs are very similar, but near the peak wavelength (465 nm), the planar ARC performs slightly better (∼0.02). At 45°, the difference between the two coatings favors the nano-ARC, as the planar ARC peak EQE drops by 0.04, whereas the nano-ARC EQE remains the same. The difference is even more evident at an angle of 60°, where the planar ARC peak EQE drops significantly by 0.12, but the nano-ARC EQE only drops by 0.01, demonstrating in practice the angle-independent operation. The numerical values for peak EQEs are shown in Table 2.

**Table 2:** Peak EQE Values at 465 nm for the GaInP Solar Cells with Different Coatings

angle of incidence	uncoated	e-Beam TiO_2_/SiO_2_	IBS TiO_2_/Ta_2_O_5_/SiO_2_/Al_2_O_3_ nano-ARC
0°	0.62	0.86	0.84
45°	0.57	0.82	0.84
60°	0.51	0.74	0.83

The drop in EQE corresponds to current density differences of 0.1 and 0.6 mA/cm^2^ favoring the nano-ARC at the angles of 45° and 60°, respectively. Both the reflectance and EQE values of the nano-ARC indicate that it should perform almost identically to the planar ARC at a normal incidence angle for the GaInP SC, which is in line with the acquired LIV results. For longer wavelengths and oblique angle operation, the nano-ARC should function clearly better than the planar reference ARC.

Using the reflectance of the different coatings and a bandgap of 1.9 eV, the nominal current densities for the GaInP SC were calculated at AM0 and AM1.5D both in an ideal case (IQE = 1; EQE = 1-*R*) and with the measured EQE, as shown in Table 3.

**Table 3:** Calculated Current Densities for Single-Junction GaInP Solar Cells with Compared ARCs Derived from the Ideal Case (IQE = 1; EQE = 1-*R*) and with Measured EQEs at Normal Incidence^a^

*J*_SC_ (mA/cm^2^)	uncoated	e-Beam TiO_2_/SiO_2_	IBS TiO_2_/Ta_2_O_5_/SiO_2_/Al_2_O_3_ nano-ARC	*R* = 0%
AM0/ideal	16.8	22.7	23.1	23.7
AM1.5D/ideal	11.2	15.2	15.4	15.6
AM0/EQE	10.9	14.6	14.6	
AM1.5D/EQE	8.1	10.8	10.8	

a The calculations use 1000 W/m^2^ for current densities calculated with measured EQE under AM1.5D.

The calculated values based on the measured EQE shown in Figure 3 and the LIV measurement results presented in Figure 2b and Table 1 are in close agreement; as for all cases, the calculated value and the measured value are within 0.1 mA/cm^2^. The existing variations in results can be linked to differences between individual SCs used in the measurements, such as the active cell area that is affected both by the used shadow mask in the contact metal deposition and the dicing precision. Based on the spectral comparison in the ideal cases, both coatings perform within 1 mA/cm^2^ of the theoretical maximum current density shown in the rightmost column of Table 3. At AM0, the nano-ARC should provide 0.4 mA/cm^2^ higher current density than the planar ARC and similarly 0.2 mA/cm^2^ higher current density at AM1.5D. Slight improvements are still possible, as the nano-ARC deviates from the ideal current density by ∼0.2 mA/cm^2^ at AM1.5D and ∼0.5 mA/cm^2^ at AM0. As the measured SCs are thinner than standalone GaInP 1-junctions would optimally be, the transmission losses cause the main difference between the ideal current densities and the ones calculated with the real EQE. Part of the difference is due to recombination losses that are neglected in the ideal case.

**Figure 4:**
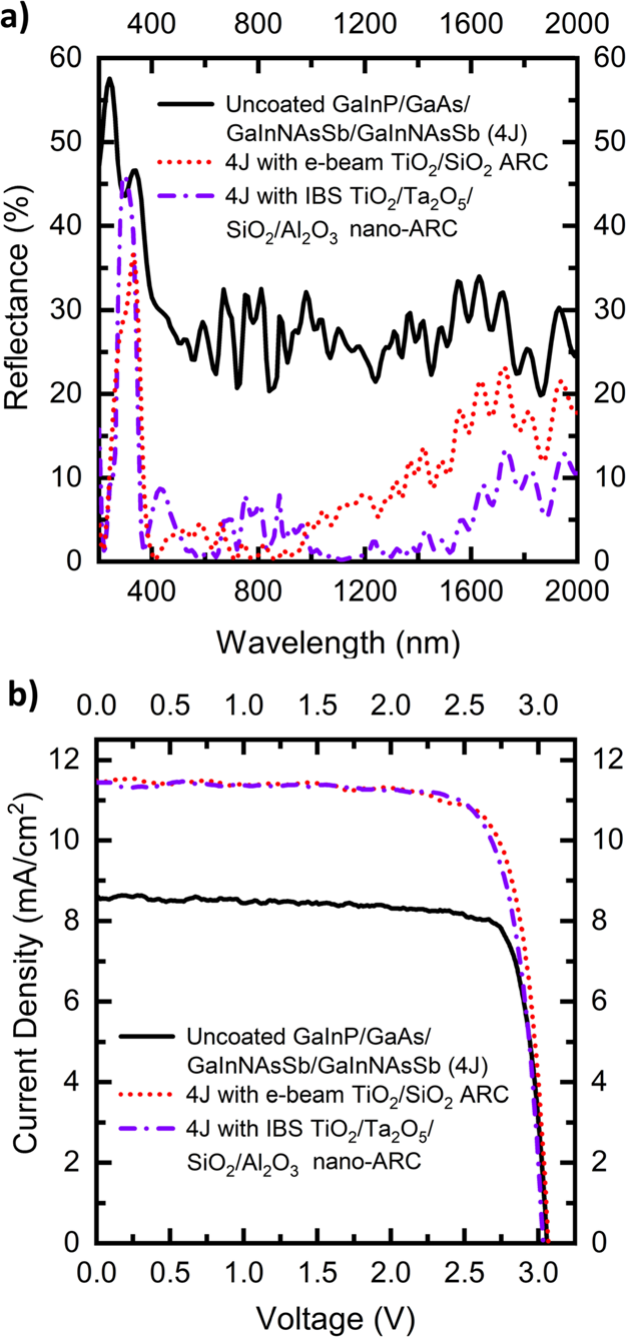
(a) URA-measured reflectance for an uncoated GaInP/GaAs/GaInNAsSb/GaInNAsSb solar cell (4J), with the conventional e-beam double-layer ARC and with the nano-ARC. (b) Measured LIV under AM1.5D (1000 W/m^2^) for the 4J solar cells without a coating, with the e-beam double-layer ARC, and with the nano-ARC.

The promising functionality on the 1J GaInP SC does not straightforwardly prove suitability for MJSCs as the current balancing, series resistance, and edge recombination scheme greatly differ between 1J and the MJSC. To this end, the ARCs were also deposited on GaInP/GaAs/GaInNAsSb/GaInNAsSb 4J. The reflectance of the MJSCs with the coatings are shown in Figure 4a. The effect of a more complex MJSC structure with additional junctions can be seen in the number of interference fringes in the reflectance measured from the bare MJSC. This complexity makes it challenging to design a balanced broadband ARC to spectrally fit the subcell current-matching requirements,^6,33^ as the average reflectance plays a smaller role than the subcell bandwidths or the MJSC overall design. Therefore, the nano-ARC structure was kept the same as for the GaInP SC, to give more comparable results.

The overall reflectance of the nano-ARC is lower than that of the planar ARC, especially at wavelengths above 1000 nm. However, the performance of the nano-ARC does not look optimal at the GaInP and GaAs bandwidths as there are several >5% interference peaks. In a case of either of the top subcells being slightly too thin and having such a high reflectance at its bandwidth, the possibility of the top cell becoming the current-limiting junction in the structure increases. To better evaluate the effects of the ARCs on the MJSC, the subcell current densities were calculated with the measured reflectance values and ideal IQE at their operation bandwidths, which are shown in Table 4.

**Table 4:** Calculated Current Densities from the *R*_AVE_ and IQE = 1 for the ARC-Coated MJSCs Presented in Different Subcell Operation Bandwidths of the 4J and in the Remaining Bandwidth that Could be Utilized with *E*_g_ ∼ 0.7 eV Subcell

*J*_SC_ (mA/cm^2^)	bandwidth (nm)		uncoated	e-Beam TiO_2_/SiO_2_	IBS TiO_2_/Ta_2_O_5_/SiO_2_/Al_2_O_3_ nano-ARC	*R* = 0%
GaInP	280–650	AM0	15.7	20.9	20.9	22.0
		AM1.5D	10.4	13.8	13.8	14.3
GaAs	650–880	AM0	12.5	17.0	16.4	17.2
		AM1.5D	10.4	14.1	13.6	14.3
GaInNAsSb (1)	880–1030	AM0	6.7	9.1	9.0	9.3
		AM1.5D	4.8	6.6	6.5	6.7
GaInNAsSb (2)	1030–1380	AM0	11.9	15.8	16.5	16.7
		AM1.5D	8.7	10.9	11.5	11.6
5th junction	1380–1800	AM0	9.6	11.3	12.6	13.4
		AM1.5D	6.2	7.2	8.2	8.7

The values in Table 4 show that the nano-ARC provides a larger current density, due to the better average reflectance than the planar ARC, only for the bottom dilute nitride junction. The reflectance of other bandwidths is of a similar scale between the nano-ARC and the planar reference, as the calculated current densities indicate, but for GaInP and GaAs junctions, it is too high and in need of optimization. This can also be seen in Table 5 as LIV values are slightly lower for the nano-ARC-coated 4J than the planar counterpart.

**Table 5:** Measured LIV Characteristics as Conversion Efficiency η, Open-Circuit Voltage *V*_OC_, Short-Circuit Current Density *J*_SC_, and Fill Factor FF under AM1.5D (1000 W/m^2^) for the 4J MJSCs as Bare, with the Planar e-Beam ARC and with the Nano-ARC

	uncoated	e-Beam TiO_2_/SiO_2_	IBS TiO_2_/Ta_2_O_5_/SiO_2_/Al_2_O_3_ nano-ARC
η (%)	21.2	27.6	27.4
*V*_OC_ (V)	3.1	3.1	3.0
*J*_SC_ (mA/cm^2^)	8.6	11.5	11.4
FF (%)	81.0	78.4	79.2

Despite the nonoptimal subcell reflectance, the nano-ARC-coated MJSC is still a functional device and no significant difference, given the statistical variations of individual cells, the limited number of samples, and the unideal spectrum of the simulator, in the electrical performance compared to the planar-coated MJSC can be observed, as is shown in Figure 4b. This would suggest that the method is suitable for MJSC ARC applications.

**Figure 5:**
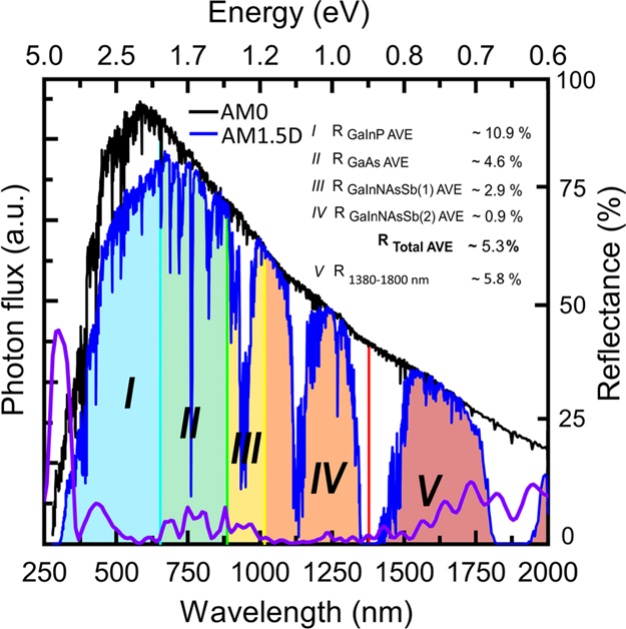
AM0 and AM1.5D^30^ spectra alongside with the measured reflectance of the nano-ARC-coated 4J GaInP/GaAs/GaInNAsSb/GaInNAsSb divided in the bandwidth of the subcells and in the bandwidth of a potential 5th junction subcell (*E*_g_ ∼ 0.7 eV). *R*_Total AVE_ shows the calculated average over a 4J bandwidth of 280–1380 nm.

The 4J GaInP/GaAs/GaInNAsSb/GaInNAsSb MJSC used in the comparison utilizes photons at the wavelengths of 280–1380 nm, which still leaves a substantial number of photons available at a bandwidth of 1380–1800 nm, as illustrated in Figure 5. The average reflectance of the nano-ARC in this fifth bandwidth is as low as 5.8%, which is 10 percentage points less than the average reflectance of the planar ARC.

With the nano-ARC, a great portion of the photons at a bandwidth of 1380–1800 nm could be utilized and at AM0 that corresponds to a current density of ∼12.6 mA/cm^2^. This is slightly lower than the current densities of the other junctions, so having an additional 0.7 eV subcell, i.e., third GaInNAsSb,^34^ would require either altering the subcell bandgaps of the current design or adding a topmost junction, such as AlGaInP,^35^ to provide nearly current-matched five or six junction SCs for space applications.

The limitations of the nano-ARC for the used MJSC subcell configuration can be overcome with structural optimization of the Al_2_O_3_ nanostructure by tuning the DIW process parameters, as done by Yin et al.^24^ and by altering the planar layer thicknesses in the multilayer configuration. The tested nanostructure was not spectrally optimized, as mainly the suitability of the method for a real MJSC device was under inspection. Our results show that the nano-ARC works also for MJSCs and there are no significant additional losses involved due to the ARC fabrication process.

## 4 Conclusions

A comparison between a nanostructured alumina multilayer ARC and a conventional planar double-layer ARC was done on a single-junction GaInP SC and 4J MJSC to assess the possible improvements related to the use of surface texturing when applied to high-efficiency MJSCs. The 1J solar cell was used for assessing the angle-dependent characteristics of the nanostructured coating, while the realistic broadband operation for MJSCs is validated using the 4J SC.

On top of the GaInP SCs, the measured reflectance over a broadband spectrum shows that for longer wavelengths, the nano-ARC performs several percentage points better than the planar ARC and the total average reflectance from 280 to 1380 nm is 2.7 and 5.5% for the nano-ARC and the planar ARC, respectively. At shorter wavelengths, the reflectance of the ARCs is of a similar scale, but due to the inward scattering of the nanotextured surface, the amount of diffused light from oblique angles is larger for the nano-ARC. This is shown in the EQE results, where the GaInP SC with the nano-ARC practically retains the same EQE level for the incident angles from 0° to 60°, whereas there is a clear drop for the EQE of the GaInP SC with the planar ARC at the larger incident angles. Better diffusion properties of the nano-ARC near the ultraviolet bandwidth and low reflectance at the infrared region point to possible performance improvements for MJSCs as well.

To address the suitability and the actual broadband operation of the nanostructured ARC on an MJSC, the same coatings were also deposited on the MBE-grown lattice-matched 4-junction GaInP/GaAs/GaInNAsSb/GaInNAsSb MJSCs. At this point, no further optimization of the coating structure was done. The LIV measurements showed that there are no evident losses caused by the nano-ARC process for the MJSC when compared to the planar coating method. The performance with the nano-ARC is adequate, but closer examination in the subcell bandwidths indicates that there is still room for improvement. In fact, the reflectance is slightly increased for all but the bottom subcell, when compared to the planar double-layer ARC. The total average reflectance over the region of operation of the MJSC is lower for the nanostructured ARC, but as the current matching limits the operation of the whole stack by the least current-producing cell, the total gain is smaller than that with the double-layer ARC. However, we believe that these shortcomings can be overcome with structural and process optimization of the nano-ARC and aim to further improve the coating performance. Also, mechanical and long-term environmental stability needs to be evaluated. As the method is lithography-free and simple, we expect to see further utilization of the nano-ARC in future MJSC architectures.
